# Supplementary material for: Early user experience and lessons learned using ultra-portable digital X-ray with computer-aided detection (DXR-CAD) products: A qualitative study from the perspective of healthcare providers
Source: PLoS One. 2023 Feb 24;18(2):e0277843. doi: 10.1371/journal.pone.0277843 (PMC9956045; doi:10.1371/journal.pone.0277843)
Supplement: S1 File — (ZIP) [file pone.0277843.s001.zip › S1 interview topic list.docx]

# S1 interview topic list:

PROJECT INFO

1. Please briefly describe your project involving the ultraportable x-ray devices and CAD software (geographical areas of focus, intervention population, main intervention, start date, end date, funding sources, etc)?
2. What are your roles in this project?
3. Which ultraportable X-ray mark and model was used in the project? (ask only if unknown)
   1. Which CAD software and version was used in the project?
   2. Was CAD software used offline or online?
   3. Did you use any other features of the CAD software (eg screen for another disease?)
4. Did you set up the x-ray in facilities or in the field?
5. How many ultraportable X-rays is the project using?
6. When did screening/triage activity first occur (*date)*?
7. What external stakeholders did you engage?
8. Did devices require registration with local authorities? If yes, with which department and what are needed?

PROGRAMMATIC SET-UP

**Ultraportable x-ray**

1. Did you use x-ray screening in parallel with symptom screening or after?
2. How many people were required on the project field team?
3. How many people were assessed with the ultraportable x-ray on an average day?
4. What positions/role did these people have (radiographer, nurse, community health worker)?
5. What safety measures do you use?
6. Did you operate on battery –power, if so, what was the battery life (hours)? Was this enough for a full day of operation without recharging?
7. Can you briefly let us know what needed to be done to install the ultraportable x-ray in the field, how long does it take normally?
8. Where is the backup data stored (physical server? cloud-based? In-country?)?
9. Did you integrate the CXR’s collected legacy systems? If yes, was this integration with national PACS? Did you build your own? or other?

**CAD**

1. Was the CAD software and ultraportable x-ray already integrated as part of a package?
2. If relevant: Have you observed any difference in performance with integrated with different x-ray machine?
3. Can you briefly let us know what need to be done to install the CAD software? What did you perform to set-up the package the x-ray and CAD package, in terms of configuration, validation and integration?
4. Did you customize the package (CAD and/or ultraportable x-ray) in any way?
5. For the CAD element, how do you select a cutoff score?
6. What factors do you think are most important for threshold score selection?

IMPLEMENTATION

**Ultraportable x-ray**

1. Did have you issue taking exposure from various type of patients – larger patient, children, etc (if yes, please explain)?
2. How was the quality of the CXR output image? Is there noticeable difference between ultraportable CXR and normal CXR quality?
3. Can high resolution DICOM be extracted directly from console or from medical imaging system?
4. Did you experience any problems with the transfer of image data from detector to console (if yes, please explain)?
5. If offline, how did you transfer data from the local monitor to the server?
   1. Were there any challenges with this?
6. Did you find the device to be portable enough for a single person to carry?
7. How often would you go on a screening mission (weekly)?
8. Did you experience any other problems to areas for improvement in use of the device?

**CAD**

1. Who use the output from the CAD for decision? (radiologists, physicians, other)? And how do they find the CAD output/ report? Do they trust the results of CAD?
2. What did patients, who received the CA output/report, find the report?

MANUFACTURER INPUT

1. What services are provided by the manufacturer?
2. Did you face any challenges with the manufacturer or is there any improvement needed to their service?
3. Did you experience any difficulty with the maintenance and warranty of the device?

TRAINING AND CAPACITY BUILDING PLANS

1. What kind of initial training did the project team receive to use the device? [who performed the training and when the training took place]
2. Did any follow-up training occur? [if yes, when, where and on what subject]
3. Did project staff have difficulty using the device after training?

USER EXPERIENCE

1. Who use the output from the CAD for decision? (radiologists, physicians, other)?
   1. And how do they find the CAD output/ report? Do they trust the results of CAD? [ask the radiologists/physician directly]
2. Do patients know their x-ray is read by CAD? What does the patient think when you tell them it is done with AI?
3. What do radiographers think about the CAD technology? [ask radiographer directly]
4. What do clinicians or radiologists think about the ultraportable x-ray device, if they have any opinion besides image quality? [ask clinicians or radiologists directly]
5. What do radiographers think about the ultraportable x-ray device? [ask radiographer directly]
6. What have you liked and dislike about the CAD product you used?
7. What have you like and disliked the ultraportable x-ray device you used?
