# Supplementary material for: Early user experience and lessons learned using ultra-portable digital X-ray with computer-aided detection (DXR-CAD) products: A qualitative study from the perspective of healthcare providers
Source: PLoS One. 2023 Feb 24;18(2):e0277843. doi: 10.1371/journal.pone.0277843 (PMC9956045; doi:10.1371/journal.pone.0277843)
Supplement: S1 File — (ZIP) [file pone.0277843.s001.zip › S2 Table 1_Initial codes_Primary, secondary, and tertiary labels .docx]

# S2 Table 1: Initial codes: Primary, secondary, and tertiary labels

| **Primary Label** | **Secondary Label** | **Tertiary Label (s) separated by comma** |
| --- | --- | --- |
| Ultraportable X-ray | Hardware | Manoeuvrability, # of devices |
|  | Set-up and connection | Image transfer from detector to console, Image transfer console to AI device, assembly duration |
|  | Varying body size |  |
|  | Radiation safety and regulation |  |
|  | Power and electricity | Battery power, external power sources, generator cycle time |
| CAD software | Internet requirement |  |
|  | Interpretation |  |
|  | Threshold score selection |  |
| CAD-DXR Package | Integration |  |
|  | Result storage | Manual, Cloud, Privacy |
| Implementation | HR |  |
|  | Training |  |
| Manufacturer Input | Maintenance | Tech-support, Machine malfunction |
|  | Importation |  |
| User Experience | Project director/ field coordinator/ technical expert/ similar |  |
|  | Clinician |  |
|  | Radiologist/ Radiographer |  |
