# Supplementary material for: Early user experience and lessons learned using ultra-portable digital X-ray with computer-aided detection (DXR-CAD) products: A qualitative study from the perspective of healthcare providers
Source: PLoS One. 2023 Feb 24;18(2):e0277843. doi: 10.1371/journal.pone.0277843 (PMC9956045; doi:10.1371/journal.pone.0277843)
Supplement: S1 File — (ZIP) [file pone.0277843.s001.zip › S3 Table 2_Relevant CFIR concepts .docx]

# S3 Table 2: Relevant CFIR concepts

| CIFR Construct | | Definition | Relevant secondary/tertiary label |
| --- | --- | --- | --- |
| Intervention Characteristics | Adaptability | The degree to which an intervention can be adapted, tailored, refined, or reinvented to meet local needs. | Battery and throughput |
|  |  |  | Internet dependence |
|  |  |  | Manoeuvrability & stability |
|  |  |  | Image quality & suitability for patients with diverse body sizes |
|  | Complexity | Perceived difficulty of implementation, reflected by duration, scope, radicalness, disruptiveness, etc. | Hardware and software integration |
|  |  |  | Interoperable data ecosystem |
| Outer Setting | External Policy | External strategies to spread interventions including policy and regulations, external mandates, recommendations, guidelines etc. | Radiation safety & regulation |
|  |  |  |  |
| Inner Setting | Readiness for Implementation- Access to knowledge and information  & Available resources | Ease of access to digestible information and knowledge about the intervention and how to incorporate it into work tasks. | Capacity building |
|  |  |  | CAD threshold score selection |
|  |  |  | Maintenance and support |
| Characteristics of individuals | Knowledge & Beliefs about the Intervention | Individuals’ attitudes toward and value placed on the intervention as well as familiarity with facts, truths, and principles related to the intervention. | Conservative application |
| Process | Executing | Carrying out or accomplishing the implementation according to plan. | Storage and Back-up of Results |
|  |  |  | Data Privacy |
